# Different age-independent effects of nutraceutical combinations on endothelium-mediated coronary flow reserve

**DOI:** 10.1186/s12979-018-0138-3

**Published:** 2018-11-22

**Authors:** Roberta Esposito, Regina Sorrentino, Giuseppe Giugliano, Marisa Avvedimento, Roberta Paolillo, Ciro Santoro, Maria Scalamogna, Mafalda Esposito, Federica Ilardi, Francesco Rozza, Giovanni Esposito, Maurizio Galderisi, Valentina Trimarco

**Affiliations:** 1 Department of Advanced Biomedical Sciences, Federico II University Hospital, Naples, Italy; 2 Interdepartmental Laboratory of Cardiac Imaging, Federico II University Hospital, Via Pansini 5, 80131 Naples, Italy

**Keywords:** Cholesterol-lowering, Nutraceutical therapy, Coronary flow reserve, Cold pressure test, Endothelial function

## Abstract

**Background:**

Some components of Nutraceuticals (NUT) such as red yeast rice and *Morus alba* have demonstrated positive effects on the endothelial function in hypercholesterolemic subjects. Our aim was to compare the effects of two different NUT combinations on cold pressure test (CPT) derived coronary flow reserve (CFR) assessed by transthoracic echo-Doppler.

**Results:**

In a randomized, single-blind study, 28 consecutive patients with a variety of cardiovascular risk factors received NUT A (LopiGLIK®: berberine, red yeast rice powder, and leaf extract of *Morus alba*) or B (Armolipid Plus®: policosanol, red yeast rice, berberine, astaxantine, folic acidandcoenzyme Q10). An echo-Doppler exam with evaluation of CFR was performed at baseline, 2 h (acute test) and 30 days after daily NUT assumption. Blood sampling for metabolic profile and platelet aggregometry was performed at baseline and after 30 days of daily NUT assumption. CFR was not significantly modified at the acute test. After 30 days, CFR improved with NUT A (*p* < 0.0001), because of the increase of hyperemic flow velocity (*p* = 0.007), but not with NUT B. CFR was comparable between the two groups at baseline but became significantly higher after 30 days in NUT A (*p* < 0.02), with a higher CFR percent variation versus baseline (*p* = 0.008). Total cholesterol and LDL-cholesterol were reduced with both NUT A (*p* < 0.001 and *p* < 0.002, respectively) and B (both *p* < 0.02), whereas platelet aggregation did not significantly change. In the pooled group of patients, after adjusting for age and percent changes of systolic blood pressure, heart rate, LDL-cholesterol and glycemia, NUT A – but not NUT B - was independently associated with CFR changes (β = 0.599, *p* = 0.003).

**Conclusions:**

LopiGLIK® improved endothelial-derived CFR, independently of the beneficial effects exerted on the lipid profile. These findings can have clinical reflections on the prevention of age-related inflammatory diseases including coronary artery disease.

**Trial registration:**

(NUTRENDO)″(ClinicalTrials.gov, NCT02969070).

## Introduction

Cardiac coronary system includes three different compartments, which are not well anatomically defined: a proximal compartment of epicardial coronary arteries, an intermediate compartment of pre-arterioles and a distal compartment of intramural arterioles, largely corresponding to coronary microcirculation [1]. Dysfunction of one of these compartments can take place even in the absence of alterations of the other compartments. Coronary system function can be tested by transthoracic Doppler echocardiography through the noninvasive assessment of coronary flow reserve (CFR), which is the maximal increase in coronary flow above its resting value for a given perfusion pressure [2]. It is well recognized that, in absence of significant stenosis of the epicardial coronary arteries, CFR represents an accurate expression of coronary microvascular function. Pharmacological agents used to induce maximal endothelium-independent hyperemia mainly include adenosine and dipyridamole. Hyperemia may even be provoked by a completely endothelium-dependent stimulus such as cold pressure test (CPT), which is performed by hand immersion in ice water for few minutes [2]. Endothelium-mediated regulation of coronary vascular tone acts through the production and release of several vasoactive mediators such as nitric oxide (NO). CPT-derived CFR is largely influenced by traditional cardiovascular risk factors and predicts future coronary events [3].

Nutraceuticals (NUT) are diet supplements that deliver concentrated forms of bioactive agents, isolated or purified from food, that are used in dosages exerting healing properties [4] and are well tolerated (hypoallergenic and digestible). NUT have shown clear beneficial effects on lipid profile [5–8]. According to recent guidelines [9–11], NUT can be used successfully either as alternatives or in addition to lipid-lowering drugs in patients with mild to moderate hypercholesterolemia.

Notably, some components of NUT such as red yeast rice (containing monacolins) and *Morus alba*, have demonstrated their positive effects on the endothelial function in hypercholesterolemic subjects [6, 12]. High cholesterol levels can reduce in fact NO’s bioavailability, allowing, therefore, onset and development of atherosclerotic lesions [13, 14]. It is conceivable that this protective effect on endothelial function could be exerted even in patients with cardiovascular risk factors other than hypercholesterolemia. Accordingly, the aim of our study was to compare the acute and 30 days effects of two different NUT combinations on CPT-derived CFR, in a population of patients with different cardiovascular risk factors, wide-ranging hemodynamic profile and variable age.

## Methods

### Study protocol and population

This is an ancillary study of the clinical trial “Effects of Nutraceutical Therapies on Endothelial Function, Platelet Aggregation, and Coronary Flow Reserve (NUTRENDO)” (ClinicalTrials.gov, NCT02969070). In particular, it is a single center, randomized, single-blind study in which consecutive patients with cardiovascular risk factors received a NUT combination (combination A or combination B) for a 30 days period. The Combination NUT A contained: berberine (531.25 mg), red yeast rice powder (220 mg, 3.3 mg monacolin K) and leaf extract of *Morus alba* (200 mg) and has been approved in Italy (LopiGLIK®, Akademy Pharma). The Combination NUT B contained: policosanol (10 mg), red yeast rice (200 mg, 3 mg monacolin K), berberine (500 mg), astaxantine (0.5 mg), folic Acid (200 mcg) and coenzyme Q10 (2 mg) and is actually approved in Italy for the control of dyslipidemia (Armolipid Plus®, Rottapharm SpA).

The study population included consecutive adult patients (age > 18 years) with cardiovascular risk factors, recruited among the staff personnel of our Department during a screening period for cardiovascular prevention. Among the hypercholesterolemic patients, we enrolled those not requiring statins or statin intolerant patients. Exclusion criteria were intolerance to NUT compounds, pregnancy and high cardiovascular risk profile, overt coronary heart disease and/or heart failure, hemodynamically significant valvular heart disease, primary cardiomyopathies, permanent atrial fibrillation, and inadequate echocardiographic images. The study was carried out following the rules of the Declaration of Helsinki, and the protocol was approved by the Ethics Committee of University of Naples Federico II (258/16). All participants gave their written informed consent.

### Procedures

A targeted clinical and familial history was summarized for each patient. All blood samples were collected from the antecubital vein between 0800 and 0900 h after an overnight fast, for the assessment of metabolic profile, and adenosine diphosphate (ADP) and non-ADP platelet aggregometry. A complete echo-Doppler exam with CPT-derived CFR of left anterior descending coronary artery was performed at baseline (i.e., at the randomization time), and repeated after 2 h (acute test) and 4 weeks after the assumption of NUT combinations (chronic test).

### Echocardiographic procedures

Echo-Doppler examinations were performed with a Vivid Seven Sound machine (GE) equipped with a 2.5 MHz phased array transducer with harmonic capability, according to the procedures of our echo lab [15] and current recommendations [16, 17].

### CPT and CFR

CPT was performed by placing the subject’s hand and distal part of the forearm in an ice water slurry for 4 min [18, 19]. Coronary flow was visualized in the distal left anterior descending coronary artery by transthoracic Doppler echocardiography with a 5 MHz shallow-focus phased-array transducer in the low parasternal long-axis cross section under the guidance of color Doppler flow mapping, according to a standardized protocol of our echo laboratory [20–22]. Doppler sample volume was placed on the color signal of the left anterior descending artery, and the characteristic biphasic flow pattern with a larger diastolic and a smaller systolic component was recorded. Attention was taken to maintaining a constant incident angle (< 30°) between coronary flow and the Doppler beam during the overall exam duration. Coronary diastolic peak flow velocities (cm/s), heart rate, and blood pressure were measured at rest and soon after the CPT at maximal endothelial induced hyperemia. CFR was calculated as the ratio of hyperemic-to-resting diastolic peak velocities (the highest three spectral Doppler signals were averaged for each measurement). Blood pressure (BP) and heart rate were determined at the beginning and the end of CPT test. Reproducibility of CPT-derived CFR measurements of our echo laboratory has been previously reported (2.0% of intra-observer variability and 4.5% of inter-observer variability of 4.5%) [21]. All images were analyzed off-line by two operators who were blind to the patients’ clinical characteristics and NUT assumption. The CFR technique operators were unaware of the NUT therapy prescribed to individual patients.

### Blood sampling and platelet aggregation

Fasting blood samples for evaluating metabolic profile and platelet aggregation were performed at baseline and after30 days of daily NUT treatment.

The measurements of glucose, total cholesterol (TC) and triglycerides (TG) (all in milligrams per deciliter), were performed by enzymatic methods (Boehringer Mannheim). High-density lipoprotein cholesterol concentration (in milligrams per deciliter) was obtained after precipitation with dextransulfate/MgCl2. Low-density lipoprotein cholesterol (LDL-C) was calculated according to the Friedewald equation [23].

Platelet aggregation test was performed according to the standards of our laboratory [24]. In particular, pharmacodynamic testing of adenosine diphosphate (ADP) and non-ADP (collagen)-induced aggregation was performed using light transmittance aggregometry LTA (model 700; Chrono-Log, Havertown, PA). Venous blood was collected into sodium citrate tubes. Platelet-rich plasma was obtained after blood centrifugation at 900 rpm for 10 min and platelet-poor plasma obtained after centrifugation of the rest of the blood at 3000 rpm for 10 min at 24 C. All measurements were performed within 2 h of sample collection. Platelet aggregation was measured as the increase in light transmission for 6 min, with the addition of ADP (20 μM/L) and collagen (2 μg/ml). The results are reported as a percentage of maximum platelet aggregation. High platelet reactivity was defined as maximum platelet aggregation > 59% (LTA 20 μM/L) [25].

## Results

We enrolled 28 consecutive patients (M/F: 18/10; age: 54.1 ± 9.4 years) with cardiovascular risk factors, randomly selected in two groups: Combination NUT A (*n* = 14) and Combination NUT B (*n* = 14). General characteristics of the pooled population and sub-analysis according to the type of NUT combination are presented in Table 1. The two groups were comparable for age, body mass index, diastolic BP and heart rate whereas systolic BP was significantly higher in NUT A (*p* = 0.034). The prevalence of cardiovascular risk factors did not differ significantly between the two groups. Fifteen of the 28 enrolled subjects (53.6%) presented elevated total cholesterol levels at baseline.

**Table 1:** Characteristics of the general population and sub-analysis according to NUT combination

Variable	Overall population (*n* = 28)	Combination A (*n* = 14)	Combination B (*n* = 14)	*p* value
Age (yrs)	54.1 ± 9.3	54.7 ± 9.8	52.9 ± 8.9	0.610
BMI (Kg/m^2^)	27.2 ± 3.3	25.7 ± 2.6	1.9 ± 0.15	0.204
Systolic BP (mmHg)	127.4 ± 10.5	124.5 ± 10.6	133.7 ± 8.2	**0.034**
Diastolic BP (mmHg)	77.7 ± 8.4	77.2 ± 7.3	78.6 ± 10.5	0.670
Heart rate (bpm)	69.2 ± 12.1	68.9 ± 12.8	69.5 ± 11.3	0.899
Arterial hypertension (n, %)	13 (46.4%)	7 (50.0%)	6 (42.9%)	0.705
Diabetes mellitus (n, %)	5 (17.9%)	4 (28.6%)	1 (7.14%)	0.139
Hypercholesterolemia (n, %)	15 (53.6%)	8 (57.1%)	7 (50%)	0.705
Hypertriglyceridemia (n, %)	4 (14.3%)	2 (14.3)	2 (14.3%)	1.00
Smoke habit (n, %)	9 (32.0%)	4 (28,6%)	5 (35.7%)	0.686
CV Familiar history (n, %)	14 (50.0%)	7 (50%)	7 (50%)	1.00

*BMI* Body mass index, *BP* Blood pressure, *CV* Cardiovascular. Boldface= statistically significant *p* value

### CPT-derived CFR

#### Acute test (Table 2)

At the CPT performed 2 h after NUT assumption, both NUT A and NUT B did not induce significant changes in patients’ CFR or hemodynamic profile (BP and heart rate).

**Table 2:** CFR at time 0 and after 2 h NUT combination intake (acute test) (t-test for paired data)

	Time 0 (*n* = 14)	Acute Test (*n* = 14)	*p* value
Combination A			
Resting Systolic BP (mmHg)	124.6 ± 6.3	123.2 ± 13.2	0.675
Diastolic BP(mmHg)	79.3 ± 6.5	78.2 ± 8.2	0.487
Heart rate (bpm)	66.1 ± 13.1	68.2 ± 12.7	0.532
Coronary flow velocity (cm/s)	20.1 ± 4.4	19..2 ± 3.4	0.281
Post CPT Systolic BP (mmHg)	122.9 ± 9.9	123.2 ± 14.4	0.944
Diastolic BP (mmHg)	74.8 ± 7.5	78.6 ± 9.3	0.157
Heart rate (bpm)	71.6 ± 15.8	72.0 ± 13.7	0.934
Coronary flow velocity (cm/s)	28.6 ± 6.3	28.7 ± 4.6	0.957
CPT-derived CFR	1.44 ± 0.18	1.49 ± 0.16	0.160
Combination B			
Resting Systolic BP (mmHg)	133.9 ± 7.8	127.2 ± 14.8	0.236
Diastolic BP(mmHg)	80.6 ± 9.5	77.2 ± 9.0	0.169
Heart rate (bpm)	69.9 ± 10.8	71.9 ± 8.5	0.487
Coronary flow velocity (cm/s)	19.3 ± 4.5	19.6 ± 4.1	0.463
Post CPT Systolic BP (mmHg)	119.4 ± 14.7	123.9 ± 19.6	0.396
Diastolic BP (mmHg)	74.8 ± 8.6	78.3 ± 11.7	0.368
Heart rate (bpm)	71.1 ± 10.3	72.9 ± 13.8	0.567
Coronary flow velocity (cm/s)	27.5 ± 5.2	27.7 ± 4.8	0.658
CPT-derived CFR	1.44 ± 0.12	1.46 ± 0.13	0.213

*BP* Blood Pressure, *CPT* Cold Pressure Test, *CFR* Coronary Flow Reserve

*30- days test* (Table 3).

**Table 3:** CFR at baseline (Time 0) and after 4 weeks of daily NUT combination intake (t-test for paired data)

Combination A	Time 0 (*n* = 14)	4 weeks CPT CFR (*n* = 14)	*p* value
Resting Systolic BP (mmHg)	124.6 ± 6.3	125.7 ± 8.9	0.865
Diastolic BP (mmHg)	79.3 ± 6.5	80.7 ± 8.0	0.391
Heart rate (bpm)	66.1 ± 13.1	71.2 ± 10.8	0.297
Coronary flow velocity (cm/s)	20.8 ± 4.0	20.4 ± 3.9	0.542
Post CPT Systolic BP (mmHg)	121.7 ± 15.8	125.7 ± 11.6	0.474
Diastolic BP (mmHg)	73.1 ± 9.1	75.5 ± 8.4	0.476
Heart rate (bpm)	69.5 ± 15.0	69.9 ± 7.7	0.521
Coronary flow velocity (cm/s)	28.9 ± 6.2	31.9 ± 6.2	**0.007**
CPT-derived CFR	1.39 ± 0.17	1.59 ± 0.14	**< 0.0001**
Combination B	Time 0 (*n* = 13)	4-weeks CPT CFR (*n* = 13)	*p* value
Resting Systolic BP (mmHg)	133.9 ± 7.8	131.2 ± 9.6	0.269
Diastolic BP (mmHg)	80.6 ± 9.5	79.4 ± 9.7	0.862
Heart rate (bpm)	69.9 ± 10.8	67.3 ± 6.1	0.351
Coronary flow velocity (cm/s)	19.3 ± 4.5	19.2 ± 3.7	0.820
Post CPT Systolic BP (mmHg)	120.8 ± 12.9	123.3 ± 14.9	0.600
Diastolic BP (mmHg)	75.7 ± 7.6	78.3 ± 10.9	0.417
Heart rate (bpm)	69.3 ± 10.1	70.3 ± 7.3	0.531
Coronary flow velocity (cm/s)	27.5 ± 5.2	28.3 ± 5.1	0.435
CPT-derived CFR	1.43 ± 0.12	1.48 ± 0.17	0.323

*BP* Blood pressure, *CFR* Coronary flow reserve, *CPT* Cold Pressure Test. Boldface= statistically significant *p* value

**Fig. 1:**
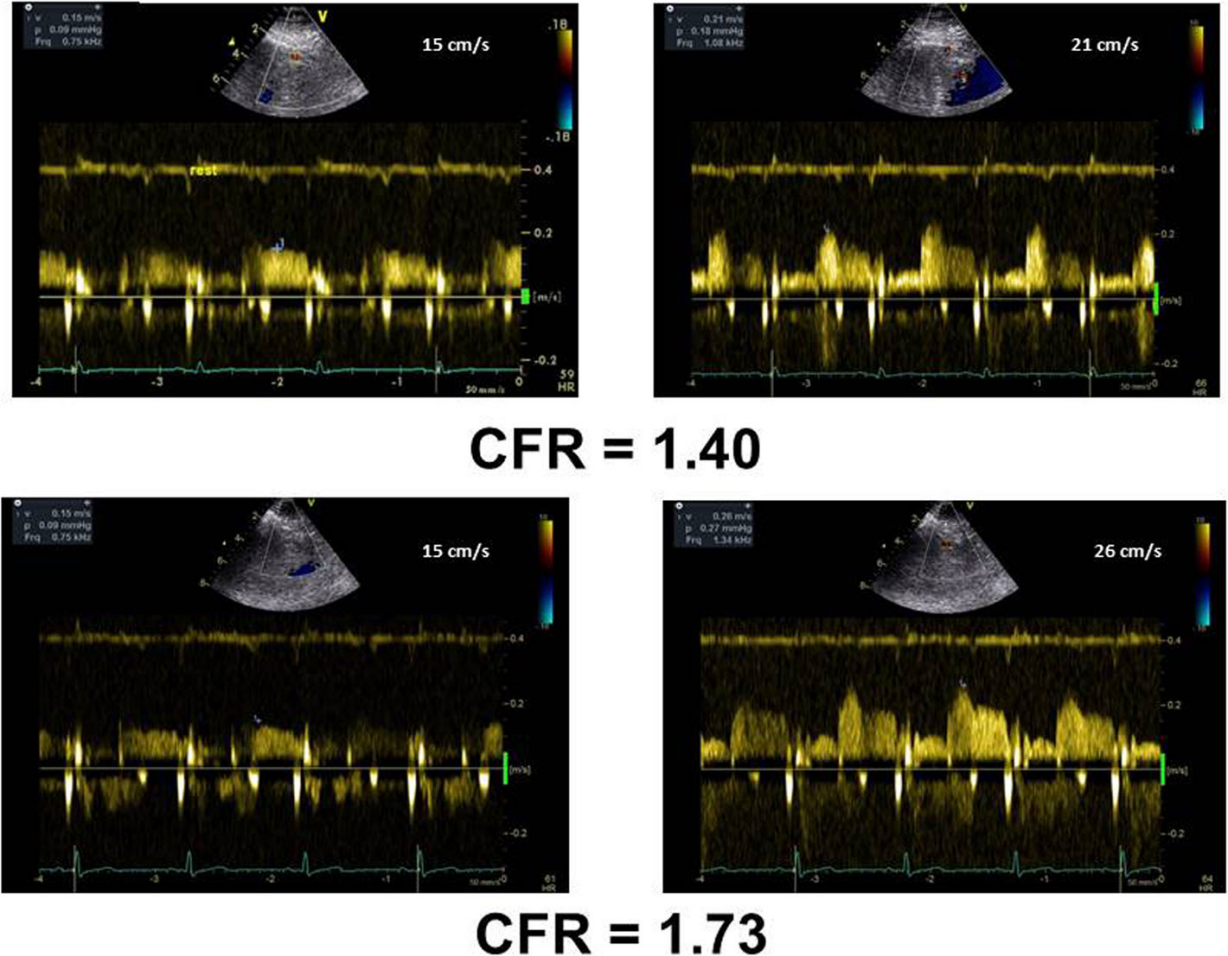
Beneficial effect of NUT A combination on CPT-derived CFR after 30 days therapy. Time 0 CFR (upper panel): baseline (left) and post CPT (right) coronary flow velocities; Combination A Time 2 CFR (lower panel): baseline (left) and post CPT (right) coronary flow velocities. CFR = Coronary flow reserve. CPT = Cold pressure test

In the NUT A group all the 14 patients repeated the CPT-CFR test after 30 days. In this group CFR substantially improved (*p* < 0.0001) in comparison with the baseline exam because of an increase of post-CPT coronary flow velocity (*p* = 0.007), whereas coronary flow velocity at rest did not significantly change. Figure 1 shows the improvement of CPT-CFR in a patient after 30 days NUT A therapy. In the NUT B group, one patient declined to repeat the CPT-CFR after 30 days because he declared an intolerance to the test (mainly, he suffered arm pain during the CPT at baseline and during the acute test). In the remaining 13 subjects, CFR was not significantly different in comparison with the baseline exam.

**Fig. 2:**
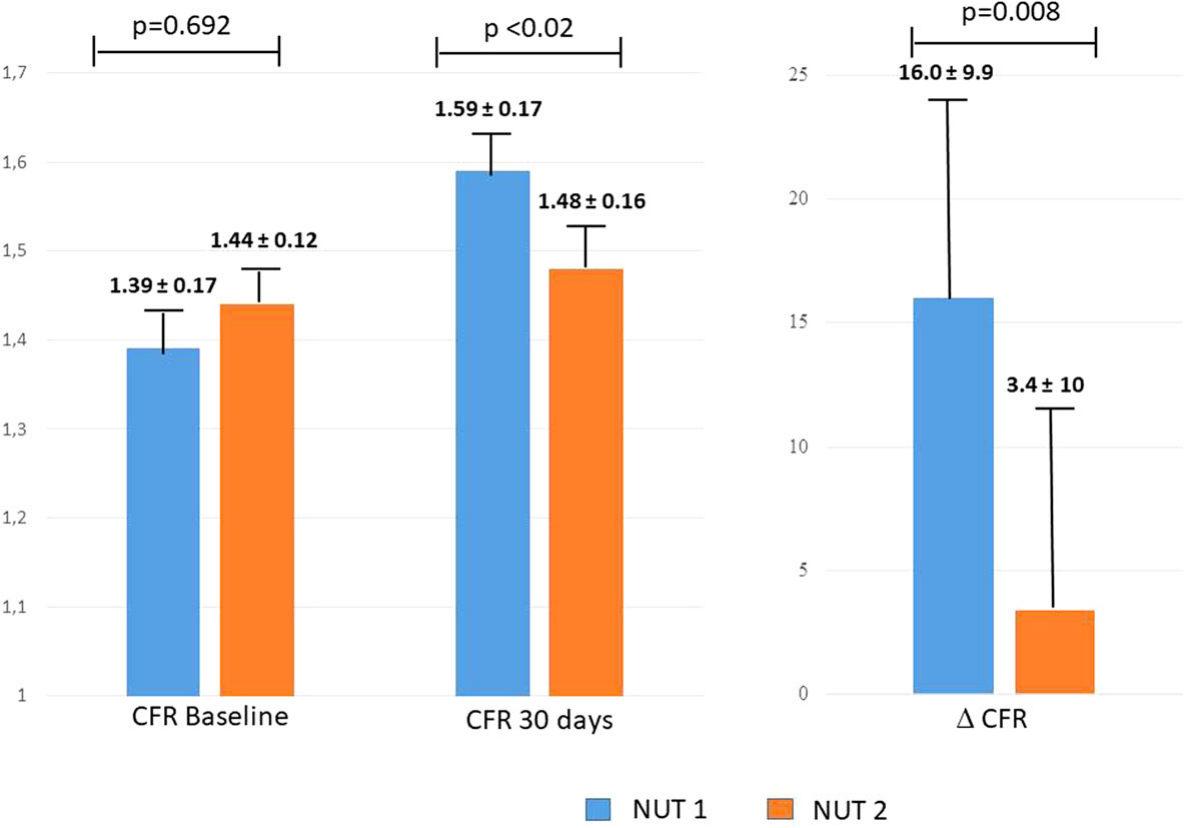
Comparison of baseline and 30 days CFR, and CFR percent changes (30 days versus baseline) in NUT A and NUT B patients

Notably, CPT-CFR was comparable between the two groups at baseline (*p* = 0.692) but became significantly different after 30 days (1.59 ± 0.14 in NUT A group versus 1.48 ± 0.16 in NUT B group, *p* < 0.02), with a higher CFR percent variation versus baseline in NUT A group (16.0 ± 9.9) than in group B (3.4 ± 10.2) (*p* = 0.008) (Fig. 2).

Also, restricting the analysis to patients without baseline hypercholesterolemia (*n* = 6), CFR was significantly improved after 30 days in the NUT A group (1.61 ± 0.11 vs. 1.41 ± 0.22, *p* < 0.02) but not in the NUT B group (*p* = 0.270).

### Blood sampling and platelet aggregation

TC and LDL-Cl were significantly lower after 30 days in comparison with baseline values in both patients assuming combination NUT A (*p* < 0.001 and *p* < 0.002 respectively) and NUT B (both *p* < 0.02). No significant difference of HDL-cholesterol, TG, glycemia and platelet aggregation assays was found in both groups at this time (Table 4).

**Table 4:** Blood assays at rest and after 4 weeks of daily NUT combination intake T-test for paired data)

Combination A	Time 0 (*n* = 14)	30 days CPT-CFR (*n* = 14)	*p* value
TC (mg/dL)	213.0 ± 39.4	197.1 ± 33.2	**< 0.001**
LDL-C(mg/dL)	138.2 ± 33.1	122.1 ± 25.9	**< 0.002**
Triglycerides (mg/dL)	130.1 ± 70.8	126.2 ± 52.7	0.828
Glycemia (mg/dL)	99.5 ± 24.3	98.2 ± 21.7	0.385
ADP(%)	66.1 ± 18.4	69.0 ± 20.1	0.700
Collagen(%)	60.1 ± 23.7	71.2 ± 25.9	0.124
ADP after insulin stimulation (%)	72.8 ± 12.1	74.5 ± 9.4	0.596
Collagen after insulin stimulation (%)	62.1 ± 26.2	67.7 ± 25.3	0.493
Combination B	Time 0 (*n* = 13)	30 days CPT-CFR (*n* = 13)	*p* value
TC (mg/dL)	209.6 ± 46.6	196.8 ± 35.6	**< 0.02**
LDL-C (mg/dL)	159.8 ± 42.9	148.5 ± 18.4	**< 0.02**
Triglycerides (mg/dL)	125.8 ± 17.7	103.6 ± 47.6	0.316
Glycemia (mg/dL)	91.6 ± 12.9	90.8 ± 7.4	0.861
ADP(%)	72.2 ± 25.4	77.3 ± 17.1	0.553
Collagen(%)	54.0 ± 39.4	69.8 ± 31.8	0.060
ADP after insulin stimulation (%)	71.5 ± 21.4	79.9 ± 11.0	0.167
Collagen after insulin stimulation (%)	55.4 ± 35.8	61.9 ± 38.6	0.156

*ADP* Adenosine diphosphate, *CPT* Cold Pressure Test, *LDL-C* Low density lipoprotein cholesterol, *TC* Total cholesterol. Boldface= statistically significant *p* value

#### Independent associations of CPT-derived CFR percent changes

In the pooled group of patients, by a multiple linear regression analysis, which adjusted for potential confounders such as age and percent changes of systolic BP, heart rate, LDL-C and glycemia, NUT A – but not of NUT B - was the only variable to be independently associated with CPT-derived CFR changes (standardized β coefficient = 0.599, *p* = 0.003) (cumulative *R*^*2*^ = 0.17, *p* = 0.03).

## Discussion

The results of this interventional, single center, randomized, single-blind study demonstrate that in subjects with a variable amount of cardiovascular risk factors and without coronary artery disease (1) both the NUT combinations significantly reduce TC and LDL-cholesterol after 30 days treatment, (2) both NUT A and B are not able to exert a positive acute effect (2 h after the assumption) on CPT-derived CFR, (3) only NUT A combination significantly improves endothelium-mediated CFR after 30 days, and (4) the beneficial effect of NUT A on CFR is independent of LDL-cholesterol changes and other covariates including age, and is not mediated by significant changes in platelet aggregation.

Previous investigations showed that treatment with NUT combinations provide a beneficial effect on the control of dyslipidaemia in patients who have mild hypercholesterolemia and/or are intolerant to statins [4–8]. Current international guidelines allow the use of NUT under these circumstances [9–11]. In a previous study, NUT A combination has already shown to be more effective than NUT B in reducing TC, LDL-C, TG and glycemia in patients with mild dyslipidaemia [6]. Our findings confirm these differences between the two NUT combinations and extend the beneficial effects of LopiGLIK® on TC and LDL-Cl also to subjects with other cardiovascular risk factors and normal TC levels (13 of the 28 enrolled subjects, 46.4%).

In the present study we tested the acute and 4 weeks effects of both NUT A and NUT B combinations on CPT-CFR. CPT is a well validated sympathetic stimulus able to induce a hyperemic vasodilation, which is totally dependent on NO endothelial release [18, 19]. In healthy subjects, *a*-adrenergically-induced CPT vascular smooth muscle vasoconstriction is counterbalanced by a subsequent ‘reactive’ endothelium-dependent hyperemic vasodilation. In pathological conditions associated with reduced NO bioavailability, the vasoconstrictor effect becomes prominent and coronary blood flow does not increase or may even decrease despite the increase of cardiac work expressed by the rate-pressure product. This methodology has been applied to transthoracic Doppler echocardiography, which allows an easy visualization of coronary flow velocities in the left anterior descending coronary artery during the test. In previous studies, we successfully used this tool to evaluate coronary endothelial function in patients with Kawasaki disease [20] and in those with mild thyroid hormone deficiency [21], two diseases in which the endothelial damage is overt. Of interest, in a subsequent study we observed that recombinant human thyrotropin administration improves CPT derived CFR in differentiated thyroid cancer patients [22], highlighting therefore the ability of this test in evaluating of pharmacologic intervention.

In the present study, both the NUT combinations failed to show significant effects on CFR in the acute test, a result which could have been expected after only two hours from the NUT assumption. Conversely, after 4 weeks therapy, NUT A – but not NUT B combination – was associated with a significant, positive effect on hyperemic coronary flow velocities and thus on CFR. To the best of knowledge, the present study is the first to demonstrate a beneficial action of a NUT combination on the endothelium of coronary arteries. These findings extend to the coronary circulation our previous observation showing the improvement of peripheral flow-mediated dilation (assessed by digital pulse amplitude) produced by a NUT combination [6]. This effect could be mainly due to *Morus alba* (white mulberry), a component originally used in the traditional Chinese medicine, present in NUT A but not in NUT B. Through its effect on endothelial nitric oxide synthase (eNOS) signalling, *Morus alba* extract seems to act as a regulator of CV system, mainly in clinical conditions characterized by eNOS impairment [12, 26]. Of note, the beneficial effects of NUT A on CFR in the present study were not associated with any kind of action on ADP platelet aggregation, thus demonstrating to be independent of rheologic profile modifications. *Morus alba* has demonstrated to significantly inhibit arterial thrombosis [27] in vivo due to antiplatelet activity tested in experiments on rats [28, 29], mainly by impairing the glycoprotein VI pathway [29]. However, this action has never been confirmed in humans.

The novelty of our findings corresponds also to the fact that the positive effect of NUT A combination on endothelium derived CFR was exerted in a population with a variable amount of CV risk factors and a wide age range (33–78 years), even in absence of hypercholesterolemia. This effect was in fact observed even in patients with normal TC levels and remained independent of the percent reduction of both LDL-cholesterol and glycemia, i.e. of the variations of individual metabolic profile. Of interest, it was independent of BP and heart rate, i.e., of rate-pressure product, whose impact on NO endothelial release is well known [18, 19]. It was also independent of age, an important finding in relation with the recognized detrimental influence of aging on coronary endothelial function in patients with metabolic diseases [30].

Our results can be explained by multiple hypotheses. *Morus alba* seems to play a fundamental role in the inhibition of alpha-glucosidase, thereby promoting carbohydrate digestion and a better post-lunch glucose profile, as well as a better insulin sensitivity [31]. *Morus alba* extract reduces BP only in wild-type mice, while it fails to provoke any hemodynamic action in eNOS-deficient mice [10].A possible anti-inflammatory effect of NUT A combination, able to improve endothelial function, could also be considered. Among patients with low-grade systemic inflammation, an oral NUT combination has already shown to significantly improve the degree of systemic inflammation and the consequent endothelial injury [32].

### Study limitations

The small sample size of the study population, mainly due to the complex protocol consisting in the repetition of three CPT tests in each patient, is the main limitation. CPT is not very well accepted since it can generate hand pain during the 4 min exposure to ice. One patient of the NUT B arm refused in fact to repeat the test after the 30 days period of daily NUT assumption. Another limitation could correspond to the absence of a definite cut-off point of normalcy for CPT-derived CFR and to the relatively small changes of the coronary flow induced by the hyperemic stimulus. However, our reproducibility of CPT-CFR has previously shown to be very good [21] with an intra-observer variability of only 2%, substantially lower than the percent increase of CFR provoked by both the NUT combinations (15 and 3% with NUT A and B, respectively).

## Conclusions

The present study demonstrates a relevant effect of a novel NUT combination, LopiGLIK®, on CFR, in comparison with another combination which does not include *Morus alba,* an extract that has shown a recognized in vivo action on endothelial function [33]. The combination of NUT with dietary counseling has already shown the ability of improving lipid profile, glycemia, diastolic BP and risk scores, and of reducing the prevalence of metabolic syndrome in patients with moderate cardiovascular risk [34]. Our findings open additional new horizons on NUT therapy in blunting or even preventing the endothelial damage and thus the atherosclerotic progression in patients with a variable amount of cardiovascular risk factors, independently of TC levels and of the effect of aging. These results could have interesting implications on the prevention of age-related inflammatory diseases including coronary artery disease.

## Abbreviations

ADP: Adenosine diphosphate
BP: Blood pressure
CFR: Coronary flow reserve
CPT: Cold pressure test
eNOS: Endothelial nitric oxide synthase
LDL-C: Low density lipoprotein cholesterol
NO: Nitric oxide
NUT: Nutraceuticals
TC: Total cholesterol
TG: Triglycerides
